# Protocols for Culturing and Imaging a Human *Ex Vivo* Osteochondral Model for Cartilage Biomanufacturing Applications

**DOI:** 10.3390/ma12040640

**Published:** 2019-02-20

**Authors:** Serena Duchi, Stephanie Doyle, Timon Eekel, Cathal D. O’Connell, Cheryl Augustine, Peter Choong, Carmine Onofrillo, Claudia Di Bella

**Affiliations:** 1 BioFab3D, Aikenhead Centre for Medical Discovery, St Vincent’s Hospital, Clinical Sciences Building, 29 Regent Street, 3065 Fitzroy, Australia; cathal.d.oconnell@gmail.com (C.D.O.); pchoong@unimelb.edu.au (P.C.); carmine.onofrillo@unimelb.edu.au (C.O.); claudia.dibella@unimelb.edu.au (C.D.B.); 2 Department of Surgery, St Vincent’s Hospital, University of Melbourne, Clinical Sciences Building, 29 Regent Street, 3065 Fitzroy, Australia; cheryl.augustine@unimelb.edu.au; 3 School of Engineering, Discipline of Electrical and Biomedical Engineering, RMIT University, 124 La Trobe Street, 3000 Melbourne, Australia; 4 University of Utrecht, Domplein 29, 3512 JE Utrecht, The Netherlands; t.m.eekel@students.uu.nl; 5 Department of Orthopaedics, St Vincent’s Hospital, 41 Victoria Parade, 3065 Fitzroy, Australia

**Keywords:** cartilage, *ex vivo* model, osteochondral unit, hydrogel-based scaffold, histological procedures, cartilage regeneration

## Abstract

Cartilage defects and diseases remain major clinical issues in orthopaedics. Biomanufacturing is now a tangible option for the delivery of bioscaffolds capable of regenerating the deficient cartilage tissue. However, several limitations of *in vitro* and experimental animal models pose serious challenges to the translation of preclinical findings into clinical practice. *Ex vivo* models are of great value for translating *in vitro* tissue engineered approaches into clinically relevant conditions. Our aim is to obtain a viable human osteochondral (OC) model to test hydrogel-based materials for cartilage repair. Here we describe a detailed step-by-step framework for the generation of human OC plugs, their culture in a perfusion device and the processing procedures for histological and advanced microscopy imaging. Our *ex vivo* OC model fulfils the following requirements: the model is metabolically stable for a relevant culture period of 4 weeks in a perfusion bioreactor, the processing procedures allowed for the analysis of 3 different tissues or materials (cartilage, bone and hydrogel) without compromising their integrity. We determined a protocol and the settings for a non-linear microscopy technique on label free sections. Furthermore, we established a clearing protocol to perform light sheet-based observations on the cartilage layer without the need for tedious and destructive histological procedures. Finally, we showed that our OC system is a clinically relevant in terms of cartilage regeneration potential. In conclusion, this OC model represents a valuable preclinical *ex vivo* tool for studying cartilage therapies, such as hydrogel-based bioscaffolds, and we envision it will reduce the number of animals needed for *in vivo* testing.

## 1. Introduction

Cartilage defects and diseases remain major clinical issues in orthopaedics. Cartilage injuries cause pain and loss of function, and if severe may result in osteoarthritis [1,2]. Regenerating healthy and long-lasting articular hyaline-like cartilage is a fundamental component of any clinical approach [3].

Biomanufacturing technologies are new strategies to address cartilage tissue repair through the generation of bioscaffolds composed of biocompatible materials and cells [4,5,6]. A major challenge for cell-based products is to fulfil critical parameters to ensure a consistent quality of the product and thereby a consistent clinical effect [7,8]. These criteria should help evaluate the performance of emerging therapies, screen for factors that will optimize their efficacy, and predict the fate of these therapies. Such qualitative and quantitative assessment features include safety and efficacy of the cells used and the type of materials implanted to generate the bioscaffold [9]. The efficacy of a tissue engineered product should be measured by biological activities and functions of the product, including relevant indication-specific key mechanisms [10]. In cartilage repair, hydrogel-based biomaterials are commonly used with tissue engineering and 3D bioprinting techniques, and the typical cells used are adult chondrocytes and Mesenchymal Stromal/Stem Cells (MSCs) [11,12,13]. 

The biological characterization of hydrogel-based biomaterials can be either modelled *in vitro* or *in vivo* in animals and ultimately in the clinical field. However, several limitations of *in vitro* and experimental models pose serious challenges to the translation of preclinical findings into clinical practice. For example, the difficulty of analyzing the interaction between the native tissue and the bioscaffolds *in vitro*, or the non-human nature of *in vivo* models and the substantial number of animals required to have a statistically significant study. Moreover, a potency assay for cartilage products should include the characteristic of the specific product transplanted and the assessment of the regeneration capacity in an environment not just similar to the native cartilage, but also mimicking the articular context where repair would occur. These evaluations can predict the cartilage regeneration capacity of a treatment before the implantation in a human patient. Although animal models have been used to assess the potency of a novel treatment [14,15,16], they pose a number of limitations. They are expensive and therefore usually only a limited number of animals are considered in a study. In relation to this, only limited numbers of time points can be evaluated per single study. The development of new treatments and the refinement of existing ones to repair cartilage defects are typically studied in either small animal models such as rodents and rabbits, or large models like sheep, goats, pigs, and horses. Cartilage defects in small animals, despite displaying a high level of complexity similar to humans, can show spontaneous self-repair [17]. This phenomenon of intrinsic repair is extremely unlikely in humans [18,19,20]. With respect to joint size and anatomy, the only clinically relevant models are large animals, but these are associated with high costs, rigorous animal husbandry and ethical issues [17,21,22]. Animals, however, can be replaced by *ex vivo* models [23]. These alternative and valuable systems allow simultaneous tests to measure cartilage repair and contribute at the same time to refining and reducing the number of animal studies performed [24,25,26]. The *ex vivo* systems are based on the generation of osteochondral (OC) units drilled out or punched out from a joint tissue, frequently the knee (stifle) condyle. *Ex vivo* OC units described in literature, despite representing a miniaturization of the entire bone and cartilage articular tissue, are usually harvested from animals such as pigs [25,27], bovines [28], and horses [29]. Human *ex vivo* models have also been proposed [30,31] but mostly to study the regenerative capacity of adult chondrocytes or MSCs without scaffold components. Very few studies use human OC plugs to test the regenerative capacity of a tissue engineered product made of hydrogel-based biomaterials. Hydrogel-based materials are becoming increasingly used in this field as a possible scaffold for cartilage repair [32,33,34]. 

One of the main technical challenges in this regard, is to analyse simultaneously both the native tissue and the implanted bioscaffold without compromising or losing the different tissue types. Also, imaging analyses are usually limited to standard histological staining on sections of a few microns’ depth, with a high risk of losing the hydrogel material during the process. This demands the use of tedious and time consuming technical procedures, which can lead to underestimating or misjudging the level of reparative/regenerative capacity of a bioscaffold in the entire OC units. What we believe is missing in the current literature is a detailed operating protocol that would facilitate researchers to handle and process the OC units, to allow standard histological analyses as well as more sophisticated imaging techniques, while maintaining the cartilage and bone tissues and the hydrogel material intact. 

In this study, we describe the culturing of a human *ex vivo* OC model and the technical imaging protocols that do not compromise the integrity of the different tissues. The *ex vivo* OC plugs were generated from condyles of donor patients, and defects were created to mimic a critical size cartilage injury. For the methodological work we used a hydrogel-based scaffold as an example and as a proof of concept. In recent work we have shown that the hydrogel formulation based on gelatin methacryloyl (GelMa) and hyaluronic acid methacrylate (HAMa), favors neocartilage formation *in vitro* in combination with adipose derived mesenchymal stem cells [35]. The GelMa/HAMa based scaffolds were cast and hardened via a photo-crosslinking reaction directly in the generated defects. The OC plugs were maintained in culture media with the help of a microfluidic perfusion bioreactor for a medium-term analysis of 4 weeks. The detailed technical protocols for histological imaging, non-linear microscopy and light sheet microscopy are presented. Furthermore, we were able to observe cartilage regeneration when the hydrogel was laden with stem cells and cast into the cartilage defect. Our *ex vivo* model will be used in the future to score in much more details the potency of GelMa/HAMa or other hydrogel-based products, in combination with cells, to repair the cartilage tissue. 

## 2. Materials and Methods

### 2.1. Generation of OC Plugs with a Critical Size Defect

OC plugs were generated from fresh femoral condyles of donor patients undergoing total joint knee replacement for osteoarthritis. The use of all human samples and procedures in this study were approved by the Human Research Ethics Committee Research Governance Unit of St. Vincent’s Hospital, Melbourne, Australia [HREC/16/SVHM/186], and all the experiments were performed in accordance with relevant guidelines and regulations. An average n = 6 of OC plugs were generated from 3 different human donors (2 females 68 and 82 years old, 1 male 76 years old) for statistical relevance. The condyle specimens were graded independently by the surgeon and two experienced researchers using the Outerbridge classification [36,37]. Areas identified as grade I and II were used to obtain the OC plugs, and regions of grade III and IV were discarded. 

**Figure 1:**
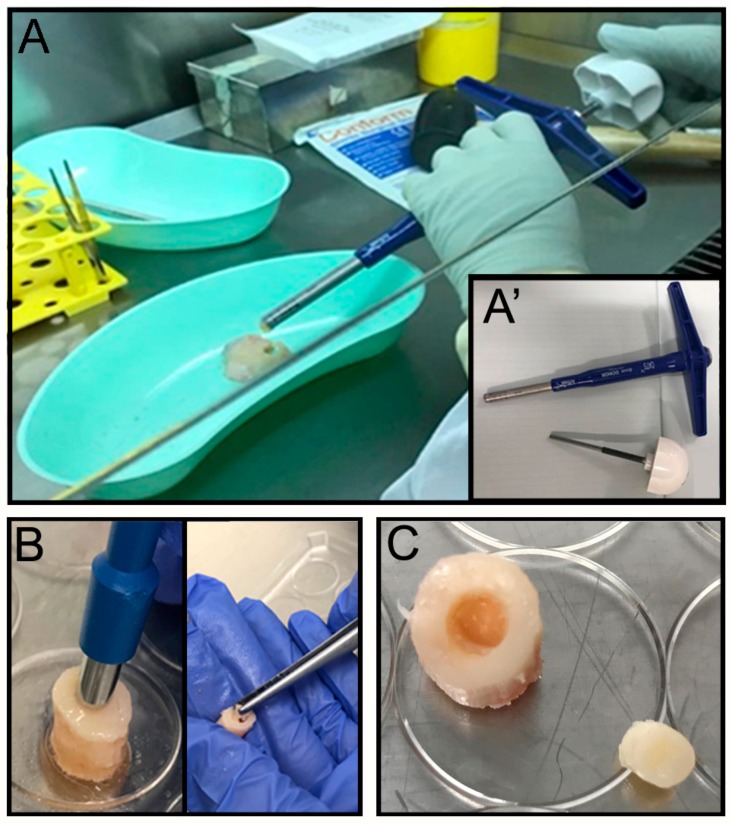
The pictures show the step-by-step procedure to obtain osteochondral (OC) plugs with cartilage defect under sterile conditions. (**A**) The OC plugs were extracted using a Φ 8 mm Arthrex OATS Biopsy bone extruder (**A’**). The focal defect was generated by using a Φ 4 mm biopsy punch (**B**) inserted into the center of the OC plug until the calcified layer right above the subchondral bone, twisted in a circular motion multiple times before removing on an angle with a ‘flick’ motion. (**C**) The removed cartilage tissue, collected inside the biopsy punch, was then discarded.

The OC plugs were extracted using an Φ 8 mm Arthrex OATS Biopsy bone extruder (Arthrex, Naples, FL, USA) under sterile conditions (Figure 1A,A’). The desired number of OC plugs were punched out with the help of a hammer, to obtain the entire depth of the condyle. Each OC plug was then properly numbered, weighed and transferred to a sterile 24 well-plate. Several washes in PBS with 100 U·mL^−1^ penicillin and 100 μg·mL^−1^ of streptomycin (Gibco, Thermo Fisher Scientific Inc., Waltham, MA, USA) were performed to remove any residual debris or blood from the surgical procedure. For each well 1.5 mL of culture media was added and samples were placed at 37 °C, 5% CO_2_ for 24 h. The next day a focal defect of 4 mm diameter was created at the center of the OC plug using a biopsy punch (Figure 1B) by twisting down to the calcified area to create a cartilaginous defect without compromising the bone underneath (Figure 1C). The focal defect within each OC plug was generated using a Φ 4 mm biopsy punch. The biopsy punch was inserted into the center of the OC plug until the calcified layer right above the subchondral bone was reached. The punch was twisted in a circular motion multiple times before removing the cartilage with a ‘flick’ motion. The removed tissue, collected inside the biopsy punch, was then discarded. The OC plugs, each with a circular defect in the middle filled with the acellular hydrogel (see Section 2.4. for details on hydrogel extrusion in the cartilage’s defect), were then transferred to a new 24 well-plate and incubated at 37 °C, 5% CO_2_, for a further 24 h before placing them in the perfusion bioreactor. The culture media for the maintenance of the OC plugs was composed by Dulbecco’s modified Eagle’s /HAMF12 (Gibco, Thermo Fisher Scientific) media supplemented with 10% Fetal Bovine Serum (FBS, Gibco), 100 U·mL^−1^ Penicillin and 100 μg·mL^−1^ Streptomycin solution (Gibco), 2 mM L-Glutamine (Gibco), and 15 mM HEPES (Gibco).

### 2.2. Culture of OC Plugs: Perfusion Culture Platform, Design of Fluidic Chambers, Fabrication and Cleaning Procedures

**Figure 2:**
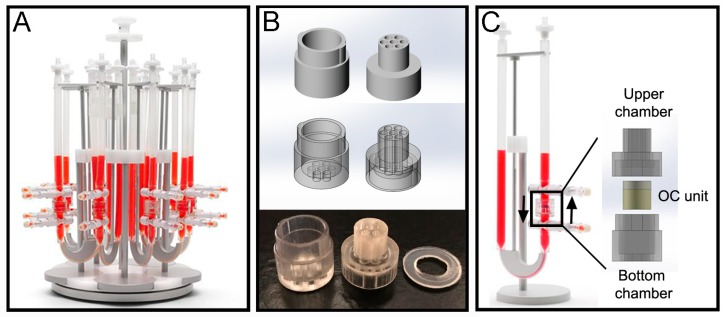
Culture of OC plugs in perfusion culture platform and custom made fluidic chamber. (**A**) Picture of the U-CUP based CellecBiotek^®^ perfusion bioreactor (Basel, Switzerland). (**B**) SolidWorks^®^ sketches and picture of the chamber fabricated via laser 3D printing, which is divided into 2 compartments: the bottom chamber includes 7 inlet channels, and the upper chamber other 7 outlet channels that go into a prolonged cylinder that can fit inside the U-CUP inner chamber without compromising the closure of the chamber. A ring of poly-dimethylsiloxane (PDMS) was clamped around the outer periphery to prevent culture media leakage during the perfusion. (**C**) The OC plugs were inserted in the fluidic chamber and then loaded into the U-CUP reservoir (black square) and the fluid allowed to perfuse bidirectionally (black arrows).

The perfusion bioreactor device from Cellec Biotek^®^ (Basel, Switzerland) (U-CUP system) was used to cultivate the OC plugs and maintain cell viability within the constructs throughout culture (Figure 2A). To provide mechanical support and prevent deformation against flow induced drag forces, the OC units were enclosed in a custom-made fluidic chamber. The chamber (Figure 2B) was fabricated via poly-jet 3D printing with an Object 30Prime Stratasys printer (Stratasys, Eden Prairie, MN, USA), using MED610 material, and were printed on high quality and glossy settings. The chamber was designed with SolidWorks^®^ (Waltham, MA, USA), and is divided into 2 parts: top and bottom. Both parts have 7 inlet channels which align when the parts are assembled. The top part has a prolonged cylinder that can fit inside the U-CUP inner chamber. Around this cylinder a 1.2 mm thick poly-dimethylsiloxane (PDMS) ring is placed to prevent leakage of media from the U-CUP system. When put together the two parts form an inner compartment with a diameter of 10 mm and height of 9 mm, into which the OC plug can be placed. Once assembled the chamber fits inside the original inner chamber provided with the U-CUP system (Figure 2C). After printing, the support material was removed manually and by soaking in 2% NaOH-1% Na_2_SiO_3_ solution, as per the manufacturer’s protocol. Once the support material was fully removed, the parts were treated to neutralize the cleaning solution first and then to sterilize them. For the neutralization procedure each part was soaked in 5% acetic acid solution for 1 min. After rinsing for 5 min in demineralized water the parts were washed in Isopropyl-ethanol, demineralized water, ethanol, demineralized water, Isopropyl-ethanol and demineralized water again, for 30 min in each solution. After the last washing step, the parts were soaked in the same OC culture media described in Section 2.1. for a week, during which the media was refreshed once.

After this cleaning protocol was completed, the parts were sterilized by soaking them in ethanol and left to dry in a biosafety hood with the UV light turned on for an hour.

The chamber was integrated within the bioreactor system as shown in Figure 2C, and 10 mL of media was used per single U-CUP and the media was changed twice a week by removing 5 mL of exhausted media and adding 5 mL of fresh media. The velocity of the syringe pump was set to a constant 3 mL/min for the entire duration of the experiment.

### 2.3. Assays of Cell Viability

To qualitatively evaluate the viability of cells in the cartilage layer right after the OC plug generation procedure, small fragments of the cartilage superficial layer were obtained with a scalpel the day after the creation of the defects in the OC plugs. A cross-sectional slice of the cartilage was cut using a scalpel (<1 mm) and was incubated with green-fluorescent Calcein-AM and red-fluorescent ethidium homodimer-1 (EthD-1) (LIVE/DEAD^®^ Viability/Cytotoxicity Kit, Thermo Fisher Scientific) solutions, according to the manufacturer’s protocol. Briefly, the working solution composed of 1 μM calcein-AM and 1 μM ethidium homodimer-1 diluted in PBS, was added to the cartilage slices and incubated at 37 °C in 5% (v/v) CO_2_ for 1 h. Samples were then imaged using an epifluorescent inverted NikonTiE microscope (Amsterdam, The Netherlands) equipped with a DS-Ri2 camera and *NIS-Elements* software (Version 4.00), using a Plan Fluor 10× DICL 0.06 NA 0.73 µm/px objective (Nikon, Amsterdam, The Netherlands). Figure panels were assembled using Photoshop software (Adobe, Version CS5.1). Three independent experiments were performed for this analysis.

To assess the viability of the entire OC plugs during time in culture, the CellTiter-Blue (Promega, WI, USA) metabolic assay was used. The solution contains resazurin, a dye which is reduced to resorufin by viable cells only. The CellTiter-Blue reagent is mixed with the culture media at a ratio of 1:4 in a total volume of 1 mL per plug. The samples were incubated for 5 h at 37 °C before the solution was removed. 500 μL of dimethyl sulfoxide (DMSO) (MP Biomedicals, CA, USA) was added to the OC plugs and incubated for a further 1 h. DMSO is a colourless, non-toxic solvent which is used to extract the dye from within the sample. After the DMSO step, the CellTiter-Blue solution was frozen at −80 °C until ready to read. CellTiter-Blue is both a quantitative and qualitative assay. The solution, after mixing with the media, is a deep blue colour due to the resazurin. However, resorufin is bright pink in colour. The degree of colour change from dark blue to bright pink will give an indication of viability of the sample. For the quantitative measurement, fluorescence is read at 550–15/600–20 nm excitation/emission, corresponding to the metabolised resorufin molecule. For the quantitative measurement, vials of the resultant CellTiter-Blue solution were thawed and 100 μL duplicates were placed into a 96-well PE OptiPlate and read using 550–15/600–20 nm excitation/emission fluorescence wavelengths in a CLARIOstar plate reader (BMG Labtech, Ortenberg, Germany). 

### 2.4. Hydrogel Material and Extrusion in the Cartilage’s Defect

**Figure 3:**
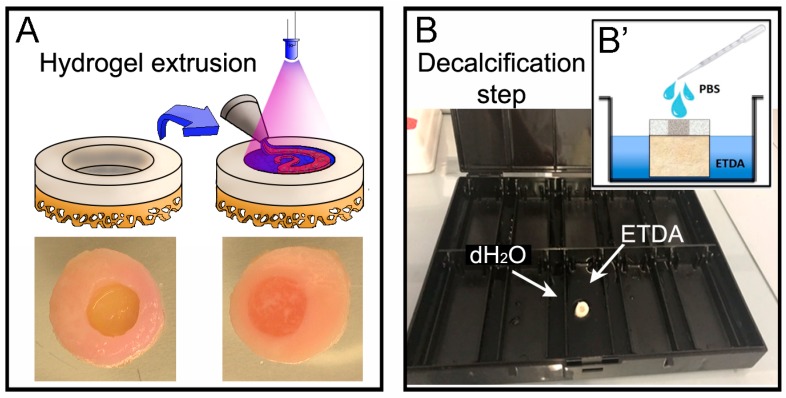
(**A**) Schematic representation of the casting procedure performed to simulate a hydrogel-based reparative approach into the cartilage defect created in the OC plugs. A gelatin-methacryloyl/hyaluronic acid methacryloyl (GelMa/HAMa) hydrogel was used as an example and extruded in to the cartilage defect and UV crosslinked to induce hardening of the material. (**B**) The picture shows the humidified chamber used to decalcify the OC plugs by submerging only the bone region with ETDA solution for a total time of 48 h. To prevent the dehydration of cartilage and hydrogels we added a drop of PBS onto the surface three times a day (**B’**).

Gelatin-methacryloyl/hyaluronic acid methacryloyl (GelMa/HAMa) was synthesized as previously described [38]. Briefly, the materials were dissolved to a final concentration of 100 mg·mL^−1^ GelMa and 20 mg·mL^−1^ HAMa (10% GelMa-2% HAMa) in sterile PBS (Sigma-Aldrich, St. Louis, MO, USA), containing 100 U·mL^−1^ penicillin and 100 μg·mL^−1^ of streptomycin (Gibco). 0.1% w/v Lithium-acylphosphinate (LAP) (Tokyo Chemical Industry Co., Tokyo, Japan) was mixed through the GelMa/HAMa and the temperature of the solutions was stabilized at 37 °C prior to preparation. A 1.5 mL syringe was used to extrude the hydrogel directly into the defect in the OC units. The hardening of the material was obtained via photocrosslinking reaction using a 365 nm UV source (Omnicure LX400+, Lumen DynamixLDGI) fitted with a 12 mm lens (25 mm focal distance) with a light intensity of 700 mW/cm^2^ at room temperature for 10 s directly on top of the casted material (Figure 3A). For the cell laden hydrogel proof of concept, we used human Adipose Derived mesenchymal Stem Cells (hADSCs) harvested from the Infra-Patellar Fat Pad (IPFP) of donor patients as extensively described in [39]. hADSCs were mixed in 10% GelMa-2% HAMa to a final concentration of 1 × 10^7^ cells mL^−1^ and extruded and harden as described above. The filled OC plugs were then maintained in the perfusion bioreactor with chondrogenic media consisting of DMEM high-glucose (Lonza, Basel, Switzerland), 100 U·mL^−1^ penicillin and 100 μg·mL^−1^ of streptomycin (Gibco,), 1X Glutamax (Gibco), and 15mMHEPES (Gibco), 1% insulin-transferring-selenium (Sigma-Aldrich), 100 nM dexamethasone (Sigma-Aldrich), 50 mg·mL^−1^ ascorbate-2-phosphate (Sigma-Aldrich), 10 ng·mL^−1^ TGFβ3 (Prepotech, Rehovot, Israel), and 10 ng·mL^−1^ BMP6 (R&D Systems, Minneapolis, MN, USA).

### 2.5. Hystological Analysis: Cryo-Embedding Procedure

The OC units were fixed with 1% Paraformaldehyde for 8 h at room temperature (RT) or overnight (O/N) at 4 °C, with gentle shaking, in a 12 well-plate. After fixation, the decalcification procedure was performed (see Section 2.7. for details). The samples were then dried well on a filter paper, put in a Cryomold Vinyl Specimen Mold container, filled with O.C.T. TM Compound (Tissue-Tek, Sakura, Leiden, The Netherlands) and flash frozen in dry ice with 2-Methylbutane embedded in liquid nitrogen. Embedded samples will be called OCT blocks.

**NOTE:** Place a 2-Methylbutan containing bowl into liquid nitrogen until ice forms at the bottom and walls of the bowl. At this point 2-Methylbutane has reached a temperature of −80 °C. Place Cryomold on the top of the frozen 2-Methylbutane and wait few seconds until all the OCT has turned white. 

The OCT blocks were then transferred directly into a box with dry ice and afterwards stored at −80 °C for at least 1 day. Cryosections were obtained using a cryostat microtome set at −25 °C/−25 °C degree for the chamber and the arm respectively, and C35 Feather Microtome blades. 10 µm thickness sections were selected to avoid folding of the sections while cutting, and then mounted onto poly-L-lysine coated glass slides (Thermo Scientific) which were stored at −20 °C before staining. 

### 2.6. Histological Analysis: Paraffin Embedding Procedure

The OC units were fixed with 10% (v/v) neutral buffered formalin Sigma #HT 501128 in a 12 well-plate for 48 h at room temperature with gentle shaking. After fixation, the decalcification procedure was performed (see Section 2.7. for details). The OC plugs were then submerged in liquid agar at 25 °C and left at 4 °C for few minutes until the agar solidified. For the dehydration (water to paraffin) the following steps were followed: 70% ethanol 2 changes 1 h each, 80% ethanol 2 changes 1 h each, 95% ethanol 2 changes 1 h each, 100% ethanol 3 changes 1 h each xylene 3 changes 1 h each, paraffin wax (56–58 °C) 2 changes 1.5 h each. The paraffin embedding was executed as follow: approximately a quarter of a large base mold was filled with paraffin wax and the sample positioned as required with tweezers. While holding the sample in place, the mold was moved to a cold plate until the wax solidified enough to hold the sample naturally. The mold was then filled with wax until it covered the entire sample. Finally, the block was left on the cold plate for 30–60 min until it was solid enough to be removed from the metal mold. The sample can be removed by banging the metal casing on the bench a couple of times to loosen the paraffin embedded sample. 

**NOTE:** If the samples are not easily removed after banging, leave on ice for longer. Remove excess wax by scraping on a heat plate. 

Sections of 10 µm were then obtained by cutting the paraffin blocks with a microtome. 

**NOTE:** Cut through the block until just before the desired section then leave the block face down in ETDA solution (arbitrarily named ETDA to differentiate it from the classic Ethylenediaminetetraacetic acid EDTA, see Section 2.7. for details) for ≈1 h. Then put block in −20 °C freezer for ≈30 min before cutting. Repeat decal and freezing process as require (i.e., the wax starts to ribbon/wrinkle, unable to cut through bone). 

The sections were mounted onto poly-L-lysine coated glass slides and left to dry O/N at 37 °C and then stored at room temperature before they were stained. 

### 2.7. Histological Analysis: Decalcification Step

This step was performed after the fixation of the OC units (either cryo and paraffin embedded). The decalcifying reagent (ETDA solution), was prepared as follow for 1 L: using a stir plate 0.7 g EDTA (0.07%) C_10_H_14_N_2_Na_2_O_8_·2H_2_O Sigma#E4884 (CAS N° 6381-92-6), 0.14 g Sodium Tartrate dibasic dehydrate (0.014%), Sigma#S4797 (CAS N° 6106-24-7) and 8 g Potassium and Sodium Tartrate tetrahydrate (0.8%), Sigma#217255 (CAS N° 6381-59-5) were added to ≈ 600 mL distilled water (dH_2_O) in a 1 L bottle. The bottle was transferred to a fume hood to add 103.45 mL of 32% HCl and the volume made up to 1 L with sterile dH_2_O. The OC plugs were placed in the largest chamber of a black slide box at room temperature. In the smallest chambers we added dH_2_O to maintain a humidified atmosphere during the process of decalcification, and this setup is herein referred to as the ‘humidity chamber’. The ETDA solution was then poured into the large chamber until the bone of the OC plug was covered and the lid was closed. The ETDA should not be covering the gel in the plug and 3 times a day we added a drop of PBS onto the surface of the cartilage/hydrogel area to avoid excessive dehydration (Figure 3B) during the process of decalcification. The ETDA solution was changed daily until the desired softness was reached (≈ 2 days). The desired softness is the end point in which there are no hard spots left in the bone, i.e., when the bone is pressed there is little or no resistance. On average this took two days, however variation between patients results in differing decalcification durations and therefore the bone was also checked when PBS was added to the gel (3 times a day). This protocol was adapted from [40].

### 2.8. Cartilage and Bone Staining of Cryo/Paraffin Sections

For the staining and analysis of cartilage and bone, three different procedures were used: histology for staining cartilage, bone and cells, immunohistochemistry for Collagen type II and immunostaining for Collagen type II and Actin. Each of these processes are explained below. 

**Histological staining**. All steps were performed in a fume hood at room temperature unless stated. For paraffin sections only we performed a rehydration (paraffin to water) step by leaving the sections at 80 °C for 20 min in an oven, followed by 3 changes in xylene 5 min each to deparaffinize. Then for both paraffin and OCT embedded sections, a detailed protocol of the staining is as follows: 100% ethanol 2 changes 1 min each, 95% ethanol 2 changes 1 min each, 80% ethanol 2 changes 1 min each, dH_2_O 2 changes 1 min each. Nuclei were stained with Weigert’s Haematoxylin (Sigma-Aldrich HT1079) placed directly on top of the slides in the sink for 5 min. 

**NOTE:** use a liquid blocker pen to draw around the sample then only one drop of Weigert’s Haematoxylin is required. 

Washes were performed in tap water until excess dye stopped leaching out of sections. Differentiation step: Acid Alcohol 1% (v/v) (70% Ethanol + Hydrochloric Acid 36.5–38%, Chem-Supply, Gillman, SA, Australia) for 2 s then dH_2_O 3 dips. Staining of bone: Aqueous Fast Green 0.05% (w/v) for 5 min. Rinse briefly in Acetic Acid 1% (v/v) (70% Ethanol + Acetic Acid Glacial, Chem-Supply) for 30 s (do not rinse). Staining of cartilage: SafraninO 0.6% (w/v) for 30 min at room temperature (do not rinse). For the dehydration steps: 95% ethanol 6 dips, 100% ethanol 2 × 8 dips; clear in xylene (Chem-Supply) 3 changes 1 min each. 

The sections were then mounted in Pertex medium (Grale HDS, Ringwood, Australia) with glass coverslips on top while trying to avoid bubbles. 

**NOTE:** transfer Pertex medium to a syringe with a stopper then leave for 15–30 min to allow all bubbles to escape before using. Let the Pertex dry out for at least 1 h at room temperature before imaging.

Samples were imaged using an inverted NikonTiE microscope (Nikon, Amsterdam, The Netherland) equipped with a DS-Ri2 camera and *NIS-Elements* software using a PlanUW 2× 0.06 NA 1.47 μm/px , Nikon objective (Nikon, Amsterdam, The Netherland). Figure panels were assembled using Photoshop software (Adobe).

**Immunohistochemistry of Collagen II.** All steps were performed at room temperature unless stated. For paraffin sections only we performed a rehydration (paraffin to water) step by leaving the sections at 80 °C for 20 min in an oven, followed by 2 changes in xylene 5 min each to deparaffinize and 2 changes in 100% ethanol for 3 min each to start the rehydration. Then to continue rehydration for both paraffin and OCT embedded sections, sections were placed in 95% ethanol for 1 min, 80% ethanol for 1 min and dH_2_O for 5 min to complete rehydration. The edges of the sample were dried and using a liquid blocker pen we restricted the staining area before rinsing with TBS 1X (TBS 10X Tris Buffered Saline: 100 mM HCl, 1.5 M NaCl pH 7.5, diluted in dH_2_O). A sufficient amount of TBST (TBS 1X + 0.1% Tween20) was added to cover the sample and let it stand for 5 min. The antigen unmasking was performed by covering the sections with freshly prepared 0.3% hydrogen peroxide (H_2_O_2_) for 5 min. Sections were washed 2 times in TBST for 2 min each. Then sections were covered with Proteinase K (ready to use reagent, DAKO #S3020) for 5 min, followed by 2 times in TBST for 2 min each wash. For the staining, the sections were placed in blocking solution (10% Normal Goat Serum in TBST) for 30 min, and after 2 times in TBST for 2 min each, washed sections were incubated with Primary Antibody (anti-collagen II, Developmental Study Hybridoma Bank DSHB #II6B3-s, 57 μg/mL) 1:250 diluted in blocking solution for overnight at 4 °C. The day after, sections were washed 3 times in TBST for 5 min each, and then left in Secondary Antibody anti-mouse biotinylated Dako #E0354, Santa Clara, CA, USA) diluted in blocking solution for 1 h. After 3 times washes in TBST for 5 min each, sections were covered with VECTASTAIN Elite ABC (Vector laboratories, Burlingame, CA, USA, #PK-6100) for 30 min. 

**NOTE:** prepare solution 30 min before use: add to 5 mL of TBST 2 drops of A + 2 drops of B and mix rapidly). After 3 washes in TBST for 5 min each, 400 µL of DAB Chromogen Substrate (Dako, Santa Clara, CA, USA was added and let it stand for 10 min to provide an acceptable staining intensity. The reaction was stopped by immersing the slides in dH_2_O. Finally, the sections were dehydrated again prior to coverslip mounting: 80% ethanol for 1 min, 95% ethanol for 1 min, 100% ethanol 2 changes 3 min each, xylene 2 changes 5 min each. Sections were mounted with coverslips using Pertex, and images taken using an inverted NikonTiE microscope equipped with a DS-Ri2 camera and *NIS-Elements* software using a PlanUW 2× 0.06 NA 1.47 μm/px Nikon objective (Nikon, Amsterdam, The Netherland). Figure panels were assembled using Photoshop software (Adobe).

**Immunostaining of Collagen II and Actin**. Cryosections were rinsed in PBS 1X and slides were dried before using a liquid blocker pen to draw a circle around the sample. Slides were put in the humid chamber and 250 µL of PBST (PBS 1X + 0.1% Triton X100) were added for 25 min. After 3 washes in PBS 1X, 250 µL of Antigen Retrieval solution (1 mg/mL HYALURONIDASE (Sigma-Aldrich, St. Louis, MO, USA) diluted in PBS 1X) were added for 30 min. After 3 washes in PBS 1X, 250 µL of blocking solution (10% normal goat serum diluted in PBST) were added for 60 min, followed by 3 washes in PBS 1X. Then 250 µL of primary antibody diluted in blocking solution (anti-collagen II (DSHB, 57 ug/mL, diluted 1:250) were added and left overnight at 4 °C. The day after, 3 washes in PBS 1X were performed for 5 min each, and 250 µL of secondary antibody diluted in blocking solution (anti-mouse IgG Alexa Fluor-647, #715-605-151, Jackson Immuno Research, 1:100) were added and left for 2 h. After 3 washes in PBS 1X, 250 µL of Phall-FITC (#P5282 Sigma—1:500 diluted in PBST) were added for 1 h, and after washes with PBS 1X, slides were mounted in Fluoromount-G (Birmingham, AL, USA). Images were taken with a NikonA1R confocal microscope using a Nikon Plan VC 20× DIC N2 N.A. 0.75 objective lens and “Scan large image” from *NIS-Elements* software tool was used to image a larger field of view.

### 2.9. Non-Linear Microscopy

Images were acquired on a fully automated, programmable, multiphoton imaging platform (Genesis^®^ 200, HistoIndex Pte Ltd, Singapore) following sectioning and mounting of 10 μm-thick cryosections washed out from OCT or 10 μm-thick paraffin sections dewaxed at 80 °C for 20 min in an oven, followed by 3 changes in xylene 5 min each. Laser excitation occurred at 780 nm. Forward-scatter two-photon excitation (TPE) and second harmonic generation (SHG) signals were detected using dedicated photomultiplier tubes for each channel. Magnification was set to 20×. TPE sensitivity was set to 0.85, and SHG sensitivity to 0.7. A bandpass filter with centre wavelength at 550 nm and bandwidth of 88 nm was set in front of the TPE photodetector. Laser baseline power was set at 0.5, and then stepped down by 60% using a set of 2 optical attenuators (0.1 OD, 0.3 OD-“Low” laser power configuration). No change was made to the polarization of the laser source, and no polarization was used on the SHG detection channel. Tissue areas were scanned at 512 × 512 pixel resolution with 2× frame averaging feature. The FibroIndex™ software (HistoIndex Pte Ltd., Singapore) was used to analyse the region of interest (ROI) in the images (https://www.histoindex.com/product-and-services/). For more detail of the procedure please refer to FibroIndex™ user manual.

### 2.10. Light Sheet Imaging: Clearing Procedure

The following protocol was adapted from [41]. The reagents were prepared according to Table 1. 

**NOTES:** 1. solutions are increasingly viscous, for ≥60% w/v fructose solutions use a glass beaker with a magnetic stirrer and lid (i.e., foil) to prevent evaporation. Gradually add D(-)-fructose completely in dH_2_O at 60 °C. After cooling to 37 °C, add α-thioglycerol to give a final concentration of 0.5% to prevent Maillard reaction (browning). 2. Do not leave SeeDB solutions at 60 °C too long (>5 h), because fructose will gradually caramelize. 3. Store SeeDB and SeeDB37 at 37 °C and all other solutions in the fridge. 

The following steps were performed before the clarification: fixation with 10% (v/v) neutral buffered formalin (Sigma-Aldrich #HT 501128) with gentle shaking overnight (tube rotator) at 4 °C. Decalcification according to the procedure in *Decalcification Step* (see Section 2.7. for details). Wash in PBS three times 10 min each. The plugs were then incubated in: 20% (w/v) fructose at 50 °C for 2 h, 40% (w/v) fructose at 50 °C for 2 h, 60% (w/v) fructose at 50 °C for 2 h, 80% (w/v) fructose at 50 °C for 2 h, 100% (w/v) fructose at 50 °C for 12 h/overnight. Finally, the plugs were soaked in SeeDB at 50 °C for 24 h and in SeeDB37 at 50 °C for an additional 24 h. OC plugs were then transferred to SeeDB and stored at 37 °C until imaging. 

**NOTE:** 1. Extension of incubation at 50 °C is not recommended. 2. Sample storage at 37 °C for many months in clearing solution has been observed to have negative effects on the hydrogel integrity and maintained tissue structural details. A small amount of sodium azide can be added to the solution for long-term storage to prevent bacterial growth. 

### 2.11. Light Sheet Imaging: Staining Procedure

A detailed protocol of the staining is here reported and for more detailed procedure please visit http://wiki.claritytechniques.org/index.php/Immunostaining.

DAY 1: Transfer the sample from SeeDB to a clean 6 well-plate and add 9 mL PBST (PBS + 0.1% Triton X100). The purpose of the washing step is to rinse out the SeeDB from inside the tissue sample. The presence of micelles inside the tissue could interfere with antibody binding during immunostaining. Incubate at RT for 24 h on a shaker/rotator plate, by changing the PBST once in the morning and once in the evening before leaving it overnight. 

DAY 2: Replace the 9 mL with fresh PBST and continue incubation with shaking for 8 h at RT. 

**NOTES:** Samples swollen in PBST will remain swollen and possibly turn cloudier than they appeared in clearing solution. This is due to refractive index differences between the tissue and PBS. TritonX is an important component in the buffer as it is a detergent, and therefore more efficient in removal of SeeDB than PBS buffer alone. Do not substitute PBS for PBST when transferring the sample from clearing solution. 

Transfer the samples in a 12 well-plate and use a volume of 2 mL in total/well for the antibody staining. Incubate with primary antibody anti Collagen type II (II-II6B3 DSHB, 57 μg/mL) diluted 1:100 in PBS + 0.2% Bovine Serum Albumin (BSA) at 4 °C on a shaker (gentle rocking) for 72 h. DAY 6: Wash sample in PBS 5 changes 30 min each. Incubate with secondary antibody anti-mouse Jackson DyLight 647 1:100 (#715-605-151, Jackson Immuno Research) diluted 1:100 in PBS at RT on a shaker (gentle rocking) for 48 h. DAY 9: Wash sample in PBS 5 changes 30 min each. Incubate with DAPI diluted 1:500 in PBS at RT on a shaker (gentle rocking) for 24 h. DAY 10: Wash sample in PBS 5 changes 30 min each. Transfer the sample in SeeDB at RT for 1 h and leave it until imaging. If the sample is more opaque, incubate the sample in SeeDB37 at 50 °C for extra 24 h.

**NOTES:** 1. Use long enough incubation times—Multiple days for incubation will likely be needed. For a 1 mm section, a good starting point is: 2-day incubation in primary antibody, 1-day wash with buffer, 2-day incubation in secondary antibody, 1-day wash with buffer. 2. Use high antibody concentrations—Start with antibody concentrations in the range of 10 μg/mL (1:100) to 20 μg/mL (1:50), and lower as needed. Begin with a 1:50 primary and secondary dilution for 1 mm blocks; adjust as needed. 3. Keep plate on a shaker for all staining and washing steps.

Images were then acquired with Light Sheet UltraMicroscope II LaVisionBiotec (Bielefeld, Germany) with a 2X CDC objective 1.25 zoom using SeeDB solution in the chamber, 639 nm wavelength laser LaserEx, 680/30 LaserEm, Andor Neo cCMOS camera, double side illumination and a refractive index of SeeDB (1.33). 

**NOTE:** it is important to choose appropriate objective lenses and right refractive index to minimize spherical aberration. 

## 3. Results

### 3.1. Generation and Culturing of OC plugs

**Figure 4:**
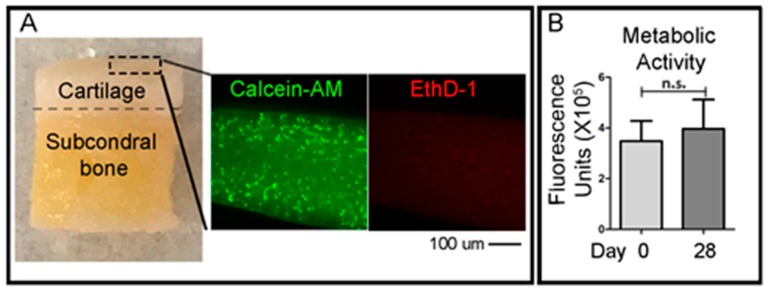
(**A**) Picture of an OC plug and representative epifluorescence images of LIVE/DEAD^®^ staining performed on cartilage slices (dotted line area in the OC plug picture). Calcein-AM (alive cells) is shown in green and Ethidium homodimer-1 (dead cells) is shown in red. Scale bar 100 um corresponds to 100 µm. (**B**) The graph shows the metabolic activity measured with CellTiter-Blue assay and fluorescence reading at day 0 and 28 days under maintenance in the U-CUP CellecBiotek^®^ perfusion bioreactor. Error bars represent standard deviation between three biological replicates. No statistical significance was found with an unpaired t test between day 0 and day 28.

OC plugs were generated from human condyles as shown in Figure 1 and described in Materials and Methods paragraph. The dimension of the defect was 4 mm diameter and 2 mm thickness for a total volume of approximately 25 mm^3^/25 μL, equal to 1.256 cm^2^ so less than <2 cm^2^ lesion, which is considered a critical size defect, meaning that a defect of this size is unable to self-repair, and it should be surgically treated with microfracture or mosaicplasty [42,43]. After the creation of the defect, the cartilage layer was analysed to verify that cell viability was not compromised by the manipulations performed. A Live and Dead assay was performed on flakes obtained with the help of a scalpel along the entire cartilage surface of the single unit. Images show that cell viability is maintained with negligible cell death after the creation of units and defects (Figure 4A). 

The OC plugs with the cartilage’s defect filled with the acellular hydrogel, were then maintained in a CellecBiotek^®^ perfusion bioreactor (Figure 2) for 28 days to first assess the stability of cell viability over time. This platform mimics the physiological *in vivo* situation, in which diffusion of signaling molecules between the cartilage and bone is only possible through the subchondral bone plate at the bone-cartilage interface and at the synovial fluid–cartilage interface. The cell viability test selected to evaluate the maintenance of the plug by time in culture, was a metabolic based test to better verify not only the presence of live cells but also their metabolism. Results show that after 28 days in our perfusion bioreactor setting, the OC plugs maintain their initial metabolic activity (Figure 4B).

### 3.2. Histological Analyses

**Figure 5:**
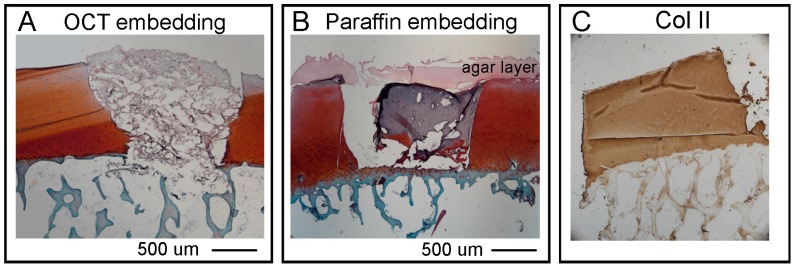
Comparison of 2 different histological embedding procedures on OC plugs cast with hydrogel material. A GelMA/HAMa hydrogel was used as an example. (**A**,**B**) Representative brightfield images of Safranin, Fast Green, H&E stainings performed on either cryo or agar/paraffin embedded samples. Scale bar 100 um corresponds to 100 µm. (**C**) Representative image of anti-Collagen type II immunohistochemistry performed on 10 µm section after cryo-embedding procedure.

As an example of a hydrogel-based material to be used for a tissue engineering reparative approach, we used GelMa10%/HAMa2%. The scaffold was cast with a syringe and UV crosslinked *in situ* to induce the hardening of the material inside the defect (Figure 3A). To evaluate the baso-lateral integration of the hydrogel scaffold it is essential to preserve the integrity of both bone (basal integration) and cartilage tissues (lateral integration). Therefore, the OC plugs were processed for histological analysis to identify the best procedure to decalcify the bone tissue while maintaining the cartilage tissue and ensuring the hydrogel material stay intact during and after the entire histological preparation. Decalcification of mineralised tissues is an essential step during tissue processing in the routine histopathology. The time required for complete decalcification, and its effect on cellular and tissue morphology, are important parameters which influence the selection of decalcifying agents. In this study, we use a decalcifying solution (ETDA) composed of both acid and chelating agents instead of a classical and well-known decalcifying agent (EDTA) [40]. To decalcify the OC units we used a humidified chamber where we submerged only the bone region of the plugs with ETDA solution for a total duration of 48 h (Figure 3B). After the decalcification step, we performed either cryo or paraffin embedding procedures and tested and compared the integrity and the quality of SafraninO, Fast Green and H&E stainings in the three tissues (bone, cartilage, hydrogel) on 10 µm sections. As shown in Figure 5, the OCT embedding partially compromises the integrity of the bone tissue while maintaining the hydrogel layer in place inside the defect (Figure 5A). However, the scaffold presents signs of dehydration. The agar and paraffin embedding allowed a better preservation of the hydrogel material despite detachment from the lateral cartilage (Figure 5B). We can hypothesize that in future applications aimed at assessing cartilage regeneration procedures, the accumulation of the chondrogenic matrix inside the hydrogel scaffold will promote baso-lateral integration, reducing the tendency for the hydrogel to detach after this technical procedure. The quality of SafraninO, Fast Green, H&E staining is comparable in the two embedding procedures and the immunoreactivity was preserved in both situations as shown by Collagen type II staining performed on cryo and paraffin sections (Figure 5C). 

### 3.3. Non-Linear Microscopy

**Figure 6:**
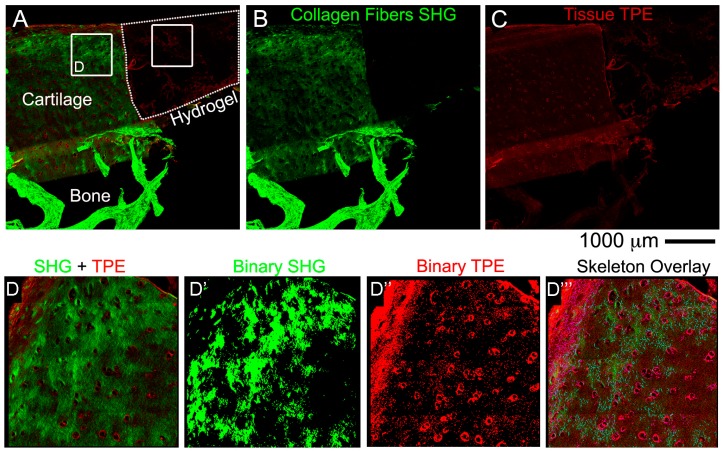
Representative image acquired with non-linear microscopy using the Genesis system on unlabelled 10 µm cryosections. The super-imposed signals of second harmonic generation (SHG) (green channel) and two-photon excitation (TPE) (red channel) are shown (**A**), while the single channels in (**B**,**C**). The 2 regions of interest (ROI)’s in the cartilage and hydrogel layers correspond to the regions where the FibroIndex™ analysis was performed. (**D**–**D‴**) Detail of the ROI of the cartilage area in **A** used to elaborate and obtain the parameters with FibroIndex^TM^ software, which were listed in Table 2 (ROI cartilage).

The immunohistochemistry analysis was complemented with non-linear microscopy, a noninvasive methodology that allows simultaneous acquisition of second harmonic generation (SHG) images of collagen fibres and two-photon fluorescence (TPE) autofluorescence images of living cells in label-free samples [44,45]. This technique can detect only mature fibrillary collagen and can be performed on the same sections generated after histological processing (either OCT and paraffin embedding) without the need of labeling with specific antibodies. Our images clearly showed collagen fibril distribution in the native OC tissues (shown in green) and cells/tissue localization (shown in red) in both cartilage and bone layers (Figure 6A–C). In the hydrogel area, as expected due to the nature of the acellular scaffold, only a background TPE signal was detectable. The FibroIndex™ software is able to detect important features in specific region of interest (white boxes in Figure 6A) to score for differences in collagen formation, maturation and localization also in the hydrogel implanted scaffold (Table 2). Figure 6D–D‴ presents the binarization and skeletonization procedure that the software uses in order to retrieve the parameters listed in Table 2. In our previous work we have demonstrated the formation and distribution of collagen fibres in stem-cell laden hydrogel materials [35], which is not shown in this paper since we used an acellular scaffold as a proof of concept for the technical protocols. 

### 3.4. Light Sheet Microscopy

**Figure 7:**
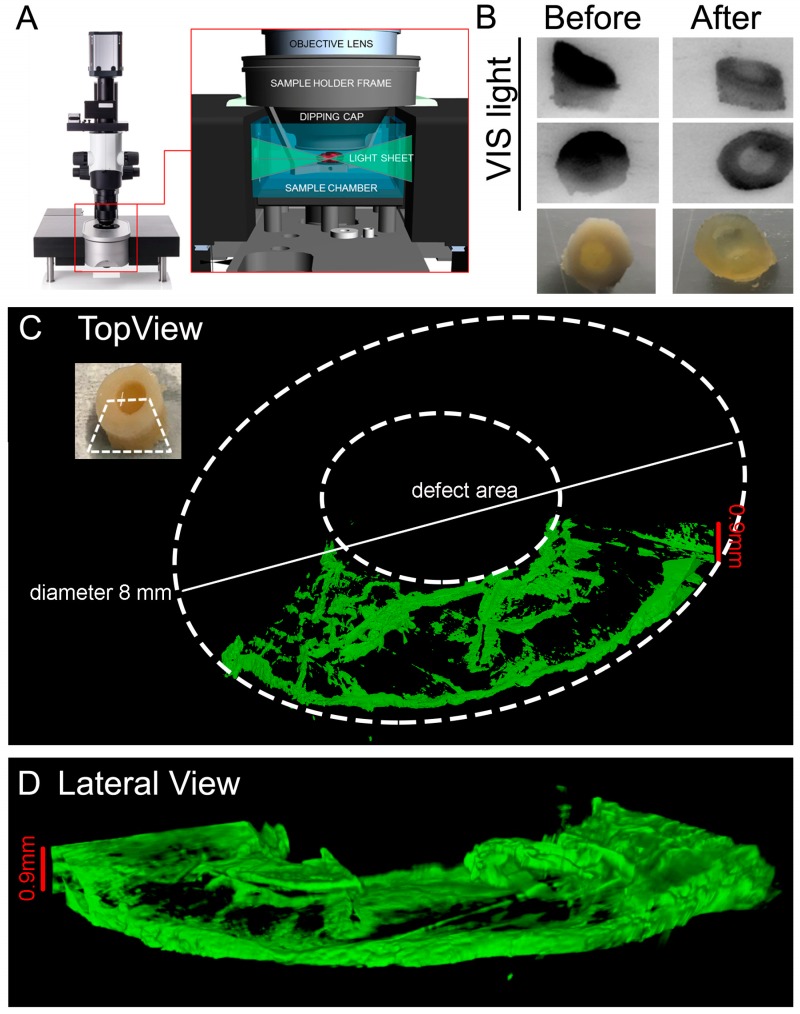
Light sheet microscopy on the whole cleared OC plugs. (**A**) Picture representing the UltraVision Ultramicroscpe used to image the OC plugs. (**B**) Representative pictures of the OC plugs with visible (VIS) light and camera before and after the clearing procedure. (**C**,**D**) Representative 3D rendered image of a section of the OC plug cleared and then stained with Collagen Type II (shown in green).

Light sheet microscopy is a fluorescence technique with an intermediate-to-high optical resolution, but good optical sectioning capabilities and high speed. In contrast to epifluorescence microscopy, only a thin slice (usually a few hundred nanometers to a few micrometers) of the sample is illuminated perpendicularly to the direction of observation (Figure 7A). To perform this type of evaluation on the entire OC unit, a level of complexity is required in the imaging procedures which is dependent on the level of transparency of the sample to be analysed. We therefore selected a protocol to allow clarification of the entire OC plugs without compromising the tissue integrity of bone, cartilage, and hydrogel layers. As described in detail in the Materials and Methods sections, the SeeDB based protocol allows us to clarify the OC units (Figure 7B) and perform light sheet imaging (Figure 7A). We used an immunostaining labelling of Collagen type II, and Figure 7C shows the region imaged to determine the feasibility of labelling post clarification. In this picture the hydrogel is excluded for simplicity since the Col-II signal in the hydrogel is undetectable, and only a portion of the OC plugs is shown to fit the sample in the imaging chamber available in our microscope (other adaptors suitable for hosting an entire sample in the chamber are commercially available). At this level of clarification of the sample, the penetration of light allowed us to resolve up to 1 mm inside the cartilage layer. Thanks to this procedure it is possible to perform multiple stainings, including nuclei labelling, without compromising tissue integrity or epitope exposure to antibodies.

### 3.5. Proof of Concept of the Regenerative Potential

**Figure 8:**
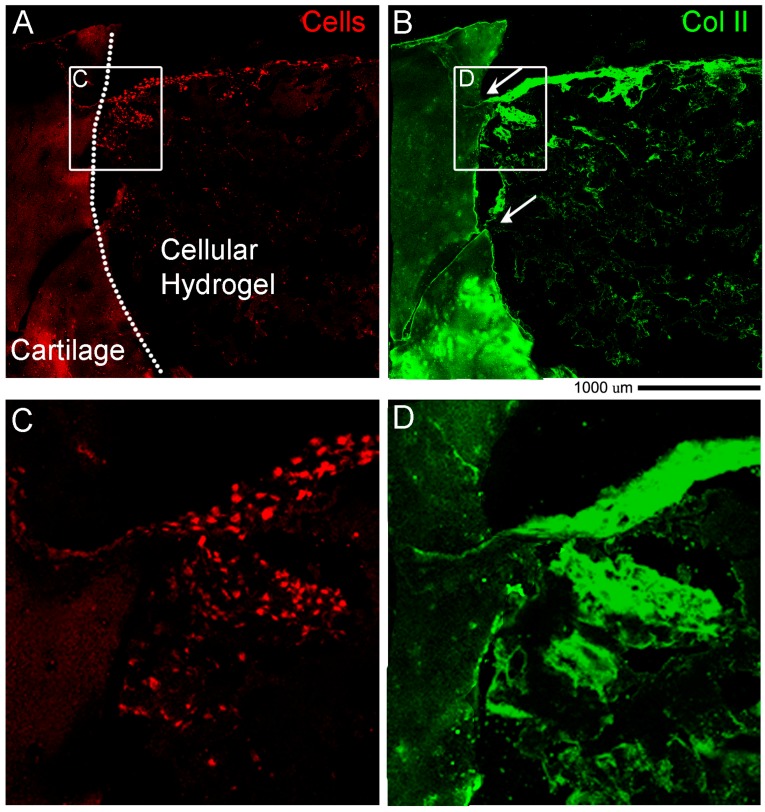
Representative fluorescent images of immunostaining performed on OCT sections of OC plugs filled with human Adipose Derived mesenchymal Stem Cells (hADSCs) cellular GelMa/HAMa hydrogel after 4 weeks of chondrogenic stimulation in the perfusion bioreactor. (**A**) Phalloidin-FITC actin staining is shown in red to mark cells in the hydrogel and in the native cartilage. (**B**) Collagen type II staining is shown in green and is clear evidence for the production and accumulation of hyaline like cartilage in the areas where the cells are aggregated. Arrows point to lateral integration areas between the scaffold and the cartilage. Scale bar 100 um corresponds to 100 µm. (**C**,**D**) Higher magnification of ROI in A and B panels.

To further strengthen the clinical relevance of our *ex vivo* model in terms of cartilage regeneration potential, we conducted a preliminary trial by extruding cell laden hydrogel into the cartilage defect and the OC plugs were maintained in chondrogenic media in the perfusion bioreactor with chondrogenic media. After 4 weeks, the plugs were processed for histological analyses and results are shown in Figure 8. With this analysis, we were able to evaluate the lateral integration of the scaffold and the distribution of hADSCs with actin staining and collagen type II production and accumulation. The areas of lateral integration, as pointed by the two arrows in Figure 8B, correspond to the region where there is a strong cluster of cells as well as the highest degree of Collagen II formation. The aggregation of cells as displayed by the Actin staining in Figure 8A confirms the viability of the cells, the ability of the cells to aggregate while undergoing the chondrogenic differentiation, as well as their tendency to move towards the natural tissue. 

## 4. Discussion

A major challenge for the translation of biomanufacturing strategies into clinical practice is the selection of appropriate and valuable *ex vivo* models. Such models allow for relevant assessment of cartilage repair while reducing the high number of animal experiments and their related costs, and to overcome the limitations of *in vitro* experimentations. These *ex vivo* models need to fulfil the following requirements: the model has to be stable, without loss of physiological properties, be viable and metabolically active for a relevant culture period of at least 4–8 weeks, which is the minimal duration of *in vivo* animal studies on cartilage regeneration therapies. Additionally, the model should facilitate monitoring of functional parameters when for instance a tissue engineered product is used to repair the damaged cartilage. One of these parameters is the baso-lateral integration within the host tissue. Several studies demonstrate that OC units represent a valuable *ex vivo* model of cartilage repair paving the way for the use of these alternative models to animal, to define and refine the translation of tissue engineering into clinical scenarios. 

In this study, we developed a culture platform that allows *ex vivo* culture, over at least 4 weeks, of human osteochondral biopsies under controlled conditions, while maintaining tissue integrity, structure and viability properties. The platform we used to cultivate the OC unit is characterized by a microfluidic chamber adapted to be hosted in a commercially available perfusion bioreactor. However, our setting differs from existing osteochondral culturing systems which employ two separated media compartments to allow culture of cartilage and bone with tissue-specific nutrients [46,47]. The microfluidic perfusion system we selected would better represent the directional fluidic passage from bone towards the cartilage and back from synovial fluid toward cartilage and bone. The platform mimics the physiological *in vivo* situation, in which diffusion of signaling molecules between the cartilage and bone is only possible through the subchondral bone plate at the bone-cartilage interface and at the synovial fluid-cartilage interface [48]. 

We created a critical size defect to mimic cartilage injuries and we used GelMa/HAMa hydrogel as a meaningful example of a scaffold to be used in combination with cells for cartilage repair, since we have already successfully shown the potency of this approach for the neocartilage formation *in vitro* [35]. 

We next presented detailed protocols to perform histological and advanced imaging analyses on the different tissues that compose the OC units, with the scope to maintain intact the bone and cartilage tissues and the hydrogel layer. The decalcification step required to process the bone tissue, was adapted from Castania et al. [40] who showed that, similarly to EDTA, the ETDA solution completely removes the calcium ions from the samples enabling easy sectioning, and in a much shorter time. Furthermore, both agents showed comparable decalcification efficiency, and similarly, they did not produce cellular, tissue or antigenicity impairments. To decalcify the OC units we used a humidified chamber where we submerged only the bone region of the plugs with ETDA solution for a total time of 48 h. We then embedded the units by both OCT and agar-paraffin protocols. The two procedures are compatible with the pre-decalcification step and they do present both advantages and disadvantages mostly related to the integrity of the hydrogel layer. Thus, to assess cartilage regeneration, we strongly recommend to perform both procedures and compare the results obtained according to the type of hydrogel used, and select the best protocol to preserve the integrity of the tissues of interest. In our hands, both cryo and agar/paraffin embedding preserved the quality of SafraninO, Fast Green and HandE stainings. The same cryo or paraffin sections, were then used to image collagen fibres via SHG by non-linear microscopy. This technique can detect only mature fibrillary Collagen and the FibroIndex™ software can easily score important features such as the amount of collagen in the tissue as well as fibre numbers, orientation and thickness. The non-linear microscopy will give important insights regarding the deposition of collagen fibres into the hydrogel and their orientation and distribution along the scaffold during maturation of the tissue. We also established a protocol to clear the OC plugs and obtain an overall view of a 1 mm thick cartilage layer by light sheet microscopy without the need for cutting the sample into thin sections. The procedure does not compromise the integrity or immunoreactivity of the tissue and will be particularly useful to verify the lateral integration of the hydrogel scaffold and the onset of cartilage repair. There are a limited number of papers describing a human *ex vivo* model however none that we were able to find with a holistic cultivation and analysis methodology with the use of a hydrogel. The novelty is that for the first time we described in detail standard and sophisticated imaging techniques while maintaining intact the cartilage and bone tissues and the hydrogel material at the same time. Another key part of the novelty of our model is the ability to decalcify in approximately two days. Finally, our work introduced a preliminary proof of concept of the regenerative potential of the *ex vivo* system. In cellular hydrogel composed of hADSC and GelMa/HAMa we observe cartilage repair in only 4 weeks of stimulation and also lateral integration. This data further strengthens the power of our system for studying clinically relevant cartilage therapies. 

## 5. Conclusions

Overall, this OC model represents a valuable preclinical *ex vivo* tool for studying clinically relevant cartilage therapies for their regenerative potential, but also for the evaluation of drug and cell therapies, or to study mechanisms of cartilage regeneration. It will undoubtedly reduce the number of animals needed for *in vivo* testing and provide the opportunity to screen the potency of engineered products and assess the regeneration capacity in an environment similar to native human cartilage. Moreover, the detailed operating protocols presented would facilitate researchers to handle and process the OC units, to allow standard histological analyses and also more sophisticated imaging techniques, while maintaining intact the cartilage and bone tissues and the hydrogel material at the same time. 

## Figures and Tables

**Table 1:** List of reagents and corresponding composition of reagents used in the clearing procedure of the OC plugs.

Reagent	Composition		
	D(-)-Fructose (≥99%)	Solvent	α-Thioglycerol (≥95%)
20% w/v	4 g	Add dH_2_O to make a total volume of 20 mL	100 µL
40% w/v	8 g		
60% w/v	12 g		
80% w/v	16 g		
100% w/v	20 g		
SeeDB	20.25 g	Add 5 mL dH_2_O	
SeeDB37	27 g		

**Table 2:** The lists of the most significant features detectable by the software FibroIndex™ is here reported. As an example of the sensitivity and performance of the software, we analyzed and compared two regions of interest (ROI, see Figure 6) respectively in the cartilage layer and in the hydrogel acellular scaffold.

Features	Description	ROI Cartilage	ROI Hydrogel
Sample Area ROI	Area of the whole tissue that was either automatically detected, or manually selected	22,438.57	22,438.57
Tissue Area	Area of tissue within the region of interest	4287.82	1890.21
Collagen Area ROI	Area of collagen within the region of interest	5714.49	0.31
Collagen Area Ratio	Relative amount of collagen compared to the total region of interest. The higher this number, the more collagen there is relative to the total region of interest.	25.47	0.00
Collagen Area Ratio in Tissue	Relative amount of collagen compared to the total tissue area. The higher this number, the more collagen there is relative to the total tissue area.	23.39	0.00
Tissue Area Ratio	Relative amount of tissue compared to the total region of interest. The higher this number, the more tissue there is relative to the total region of interest.	19.11	8.42
Collagen Fibres’ Number	Number of fibres per square millimeter	2782.60	1.00
Collagen Fibres’ Mean Thickness	Fibre mean thickness in micron	4.46	N.A.
Collagen Fibres’ Mean Length	Fibre mean length in micron	12.63	N.A.
Collagen Fibres’ Mean Straightness	Fibre mean straightness	79.71	N.A.
Collagen Fibres’ Orientation Variation	Measure of the variation of the orientation from the collagen fibres orientation distribution. A low value means fibres tend to be oriented in the same direction. 100% means the fibres’ orientation variation is maximized. i.e., fibres orientation is scattered	44.52	N.A.
Collagen Reticulation Index	A measure of how many collagen fibre branches there are within the entire length of the collagen network	4.17	N.A.
